# Supplementary material for: Oo-site: A dashboard to visualize gene expression during Drosophila oogenesis suggests meiotic entry is regulated post-transcriptionally
Source: Biol Open. 2022 May 17;11(5):bio059286. doi: 10.1242/bio.059286 (PMC9148541; doi:10.1242/bio.059286)
Supplement: Supplementary information [file biolopen-11-059286-s1.pdf]

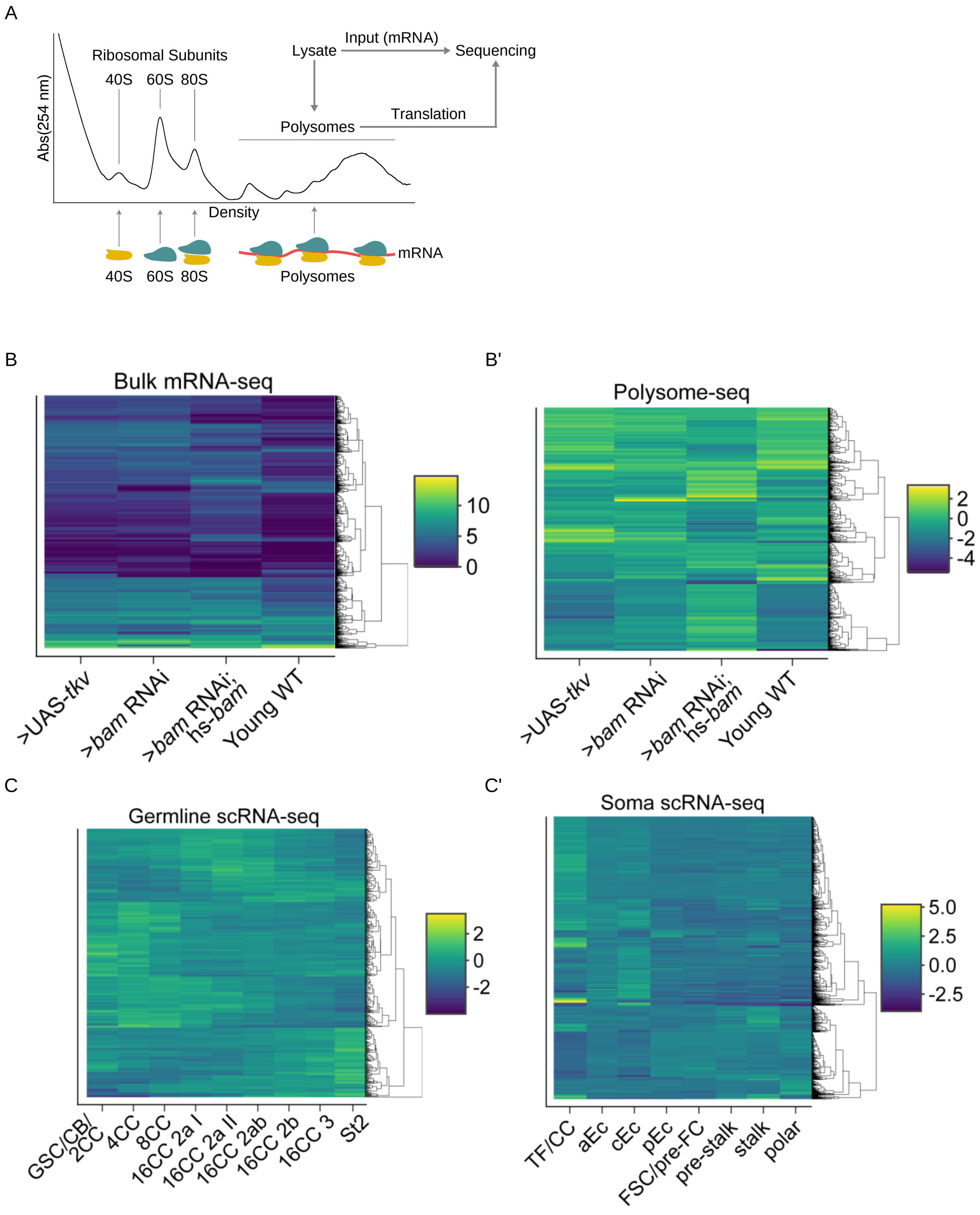

Biology Open • Supplementary information

Fig. S1. Sequencing strategy and clustered heatmaps of differential expression, related to Figure 1

(**A**) Schematic of strategy used to obtain input mRNA samples and matched polysome-seq libraries of ovaries genetically enriched for developmental milestones. (**B-B'**) Clustered heatmaps of (B) bulk RNA-seq and (B') $\log_2$(TE) from polysome-seq of the developmental milestones indicated on the X-axis. Each row in the heatmap indicates a gene that is differentially expressed in at least one of the milestones compared to all others in a pairwise fashion. Color scale denotes average relative expression. (**C**) scRNA-seq of early germline cells and (**C'**) scRNA-seq of somatic cells in the germarium, the clusters identified from scRNA-seq are denoted as follows: somatic terminal filament and cap cells (TF/CC), anterior escort cells (aEC), central escort cells (cEC), and posterior escort cells (pEC), follicle cells and pre-follicle cells (FSC/pre-FC), stalk cells (stalk), and polar cells (polar). X-axis denotes cell-type and each row in the heatmap indicates a gene that is differentially expressed in at least one of the cell-types compared to all others in a pairwise fashion.

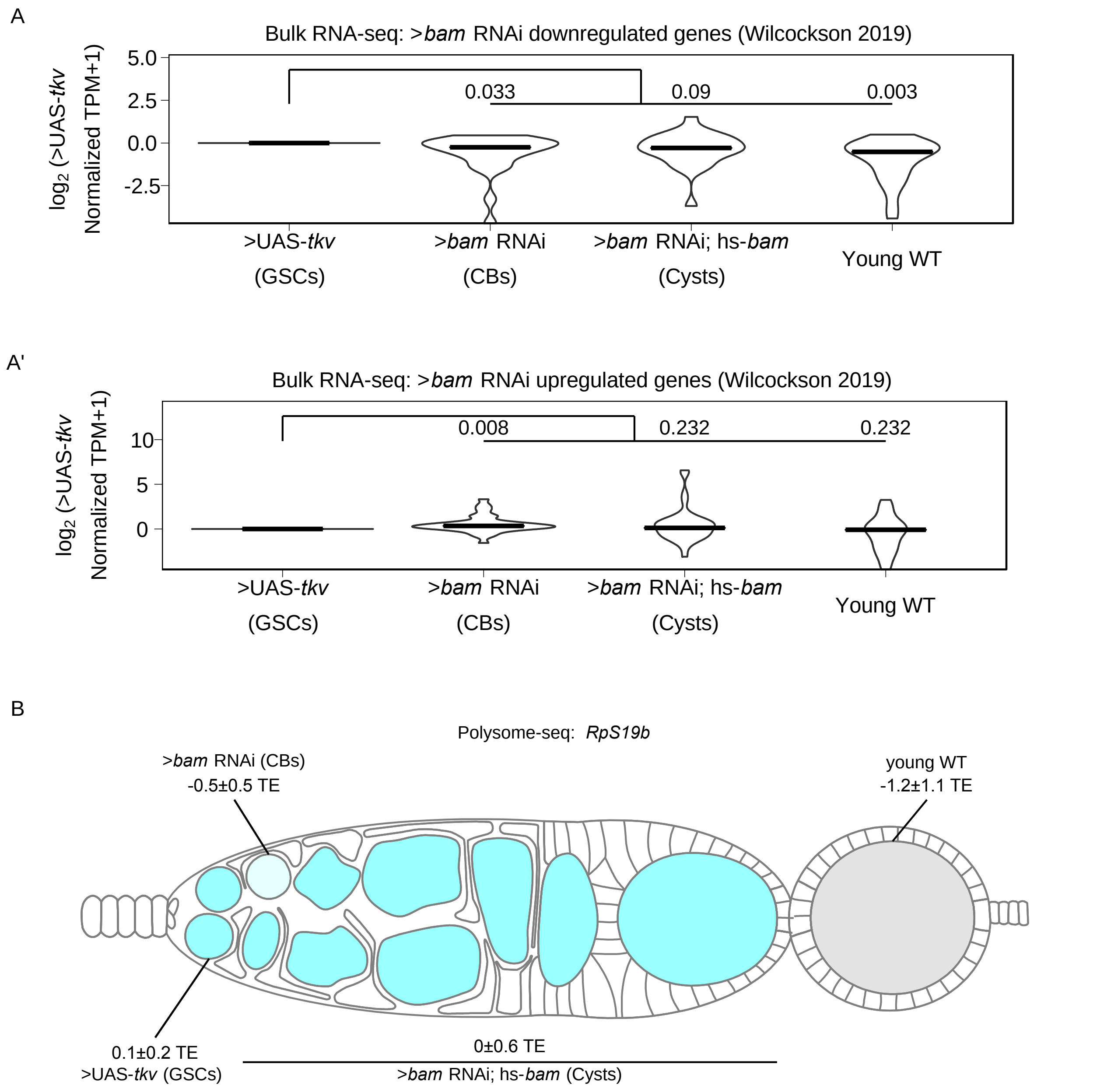

Fig. S2. Bulk RNA-seq recapitulates previously observed expression patterns of gene expression, related to Figure 2

(**A-A'**) Violin plots of expression from bulk RNA-seq of genes 2-fold or more (A) down or (A') upregulated in bam RNAi germline cells compared to UAS-TKV overexpressing germline cell with a p-value < 0.01 over germline development from Wilcockson *et al.* demonstrate that bulk RNA-seq identifies similar trends in gene expression compared to the FACS based method

Biology Open • Supplementary information

employed by Wilcockson *et al*. Values above plots represent Holm-Bonnferroni adjusted p-values from a Welch's t-test between the indicated genotypes. (**B**) Visualization of expression of *RpS19b* over germline development from polysome-seq data. Color indicates TE and values indicate the $\log_2$ mean TE±standard error *RpS19b* TE is relatively consistent during early oogenesis and decreases in the egg chambers.

Biology Open • Supplementary information

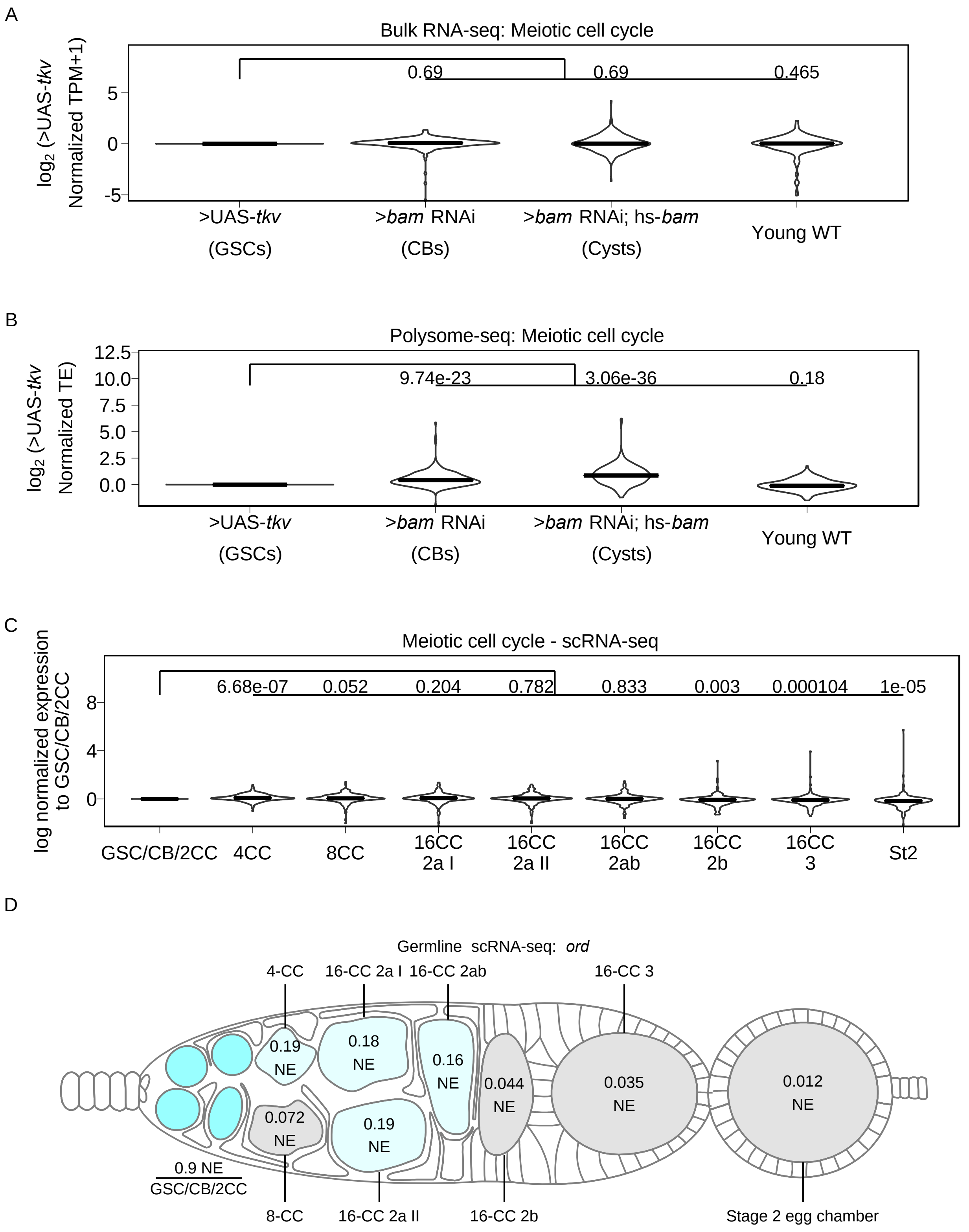

Biology Open • Supplementary information

Fig. S3. Genes involved in meiotic cell cycle, including *ord,* may be controlled post-transcriptionally, related to Figure 4.

(**A**) Violin plots of gene expression from RNA-seq of genes in the GO-term category meiotic cell cycle. No significant overall change occurs to expression of these genes at any of the developmental milestones compared to GSCs. Values above plots represent Holm-Bonnferroni adjusted p-values from a Welch's t-test between the indicated genotypes. (**B**) Violin plots of TE from polysome-seq of genes in the GO-term category meiotic cell cycle. Overall TE increases in CBs and cysts significantly compared to GSCs indicating that meiotic entry may be partially controlled post-transcriptionally. Values above plots represent Holm-Bonnferroni adjusted p-values from a Welch's t-test between the indicated genotypes. (**C**) Violin plot of expression of genes in the GO category "meiotic cell cycle" from scRNA-seq. Overall expression of these genes increases in CBs, cysts, and young-WT ovaries compared to the GSC/CB/2CC cluster. Values above plots represent Holm-Bonnferroni adjusted p-values from a Welch's t-test between the indicated genotypes. (**D**) scRNA-seq data indicate that the mRNA level of *ord* is highest in the GSC/CB/2CC cluster but remains relatively consistent in its expression starting in the 4-CC through 16-CC 2ab clusters and is dramatically decreased in early egg chambers. Color and values indicate the normalized expression of *ord* in each given stage.
